# The effect of acute pain on executive function

**Published:** 2018-08-01

**Authors:** Jenna Morogiello, Nicholas G. Murray, Tamerah N. Hunt, Brandonn S. Harris, Brian J. Szekely, George W. Shaver

**Affiliations:** ^1^Waters College of Health Professions, Department of Health and Kinesiology, Georgia Southern University, Statesboro, Georgia, United States; ^2^School of Community Health Sciences, University of Nevada, Reno, Nevada, United States

**Keywords:** Musculoskeletal, cognition, pain, neuropsychology, executive function

## Abstract

**Background and Aim:**

Executive functions are high-level cognitive processes that allow a person to success-fully engage in an independent and self-fulfilling life. Previous literature indicates that chronic pain can affect executive function, but there are limited studies that investigate the effect of acute pain on executive function. The purpose of this study was to determine if acute pain affects executive function in recreationally active indi-viduals who sustained a musculoskeletal injury.

**Methods:**

Twenty-four recreationally active participants who presented with acute pain following a muscu-loskeletal injury underwent a neuropsychological battery within 72 hours of injury. Follow up testing occurred within two weeks from the initial testing session when participants were pain free. Pain intensity was measured using the Visual Analog Scale (VAS). The neuropsychological battery consisted of the following tests: Digit Span (DS), Rey Auditory Verbal Learning Test (RAVLT), and Trail Making Test B (TMT-B). The DS was bro-ken into two separate scores, the RAVLT four scores, and TMT-B one score. Seven paired samples t-tests were conducted using an adjusted alpha level of 0.007.

**Results:**

Participants had significantly improved scores when pain free in DS forwards (*p* < 0.007) and TMT-B (*p* < 0.007). No significant difference was observed for the DS backward (*p* = 0.023), RAVLT A1 (*p* = 0.563), RAVLT sum A1 to A5 (*p* = 0.953), RAVLT A6 (*p* = 1.0), RAVLT recognition list A (*p* = 0.009). These results suggest that immediate recall and complex attention may be diminished in individ-uals who experience acute pain due to a musculoskeletal injury.

**Conclusions:**

Results from this study suggest acute pain from musculoskeletal injuries may disrupt executive function.

**Relevance for patients:**

Patients should be aware that there may be cognitive changes after a musculoskeletal injury. Knowing which cognitive domains may be impaired during acute pain could impact clinical practice and further benefit patients suffering from pain and its associated symptoms.

## 1. Introduction

The Center of Disease Control (CDC) estimated an average of 8.6 million recreation and sport related injuries per year from 2011-2014 [1]. Although there are many health benefits of liv-ing an active lifestyle, injury rates are higher among those who participate in sports or who engage in exercise [2]. Pain due to an injury has been suggested to not only affect physical perfor-mance, but also mental performance. Unfortunately, there is no universal method to treating or managing pain, leaving it as an increasingly significant public health concern. As of 2012, the total estimated annual cost in the United States due to pain ranges from 560 billion to 635 billion, straining the nation’s already bur-dened healthcare system and economy [3]. It is also worth not-ing that in the past 20 years there has been an extreme increase in therapeutic opioid consumption and abuse, with the United States having the highest consumption of narcotics worldwide [4]. Pain is tremendously subjective, influences individuals in a wide variety of ways, and can be managed using a myriad of approaches [5]. Although many individuals can perform ade-quately and/or maintain activities of daily living while experi-encing acute or chronic pain, research suggest that pain may di-rectly influence mental processes [6]. These mental processes may be involved in initiating and maintaining smooth information processing within the central nervous system [7–9].

These processes, better known as executive functions, allow one to plan and direct purposeful and flexible behavior [7]. Ex-ecutive functions provide the capacity to modify thoughts and behaviors in order to respond to a similar situation differently. If these functions are impaired, an individual may lack self-control, become irritable, and lack focusing and planning abil-ity [7]. Because executive functions are considered to be higher order thinking processes, impairments can decrease the quality of life of those who suffer from these deficiencies [10,11]. Ex-ecutive functions and cognitive functions work concomitantly; if executive functions are impaired, then cognition may be af-fected. Executive function differs from cognition, which is pri-marily involved with the input, storage, processing and output of information yet both are actively impaired in the presence of pain [7,12].

Musculoskeletal injuries commonly affect active popula-tions that engage in recreational or competitive sports and are often classified as acute. Acute musculoskeletal injuries are ones that typically result in a loss of playing time [13,14] but both in-jury severity and pain typically resolve within 2-3 weeks [15,16]. The pain experienced from musculoskeletal injuries ranges from mild discomfort to severe depending on the type of injury, the so-matic interpretation of the pain, and the pain tolerance threshold of the individual. Whether acute or chronic, if pain is present re-search indicates that executive functions may be impaired [7,17– 20]. Specifically, a reduction in attention, processing speed, and psychomotor speed is frequently noted [7]. These pain-induced alterations can also appear within higher order cognitive pro-cesses (executive functions). Since the prefrontal cortex is re-sponsible for both executive functions and encoding pain, this can lead to an uneven distribution of neuronal resources when both processes are simultaneously active [21]. Therefore, if an individual is experiencing pain his/her neural resources may be primarily devoted to pain processing, leaving less resources to dedicate to executive functions [22]. Because these processes may rely on21 overlapping networks, it is not surprising that ex-ecutive functions can be altered when pain is present [21,22].

Research involving chronic pain and musculoskeletal in-juries are extensive, however, very little investigations have measured the effect of acute pain on executive functions [12]. Hutchinson et al reported that NCAA collegiate athletes who had a current musculoskeletal injury performed worse than healthy controls on the match to sample subtest of the Neuropsycholog-ical Assessment Metrics (ANAM) [13]. These results suggest that an athlete’s thinking abilities and memory may be impaired when in the presence of a musculoskeletal injury. It is theorized that either the negative emotional state of being “sidelined” from the injury or the pain may have caused the altered cognitive state [13,23]. Both mechanisms can explain the decline in the exec-utive function of these athletes; however, neither were investi-gated nor reported on. These findings should be explored further as the presence of pain following an acute musculoskeletal injury may impede classroom and/or work performance. Furthermore, there are not many studies that examine the recreationally ac-tive population, which is a shame since most results based on the athletic population cannot be generalizable to the public.

Therefore, the purpose of this study was to explore if the recreationally active who presented with acute pain due to a mus-culoskeletal injury would have impairments in executive func-tions as measured by the Digit Span, Rey Auditory Verbal Learn-ing Test, and Trails Making Test-B as compared to a non-pain state. It was hypothesized that a difference in all neuropsycho-logical testing scores would be present among participants expe-riencing acute pain from a musculoskeletal injury compared to their testing scores when they were not in acute pain but would subside once the pain was reduced.

## 2. Materials and Methods

### 2.1. Participants

**Figure 1:**
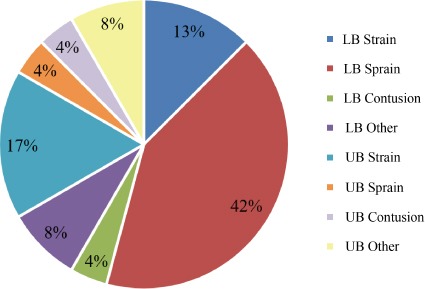
Breakdown of musculoskeletal injuries by location and type (n = 24). Descriptive injury data based on a clinical diagnosis by an athletic trainer (ATC). LB: Lower body, UB: Upper body. LB/UB Other: injuries to the lower and upper body that were not clinically diagnosed as a sprain, strain, or contusion.

Twenty-four (22 ± 2 years; 9 female, and 15 male) indi-viduals were included in this study (see Table 1). Of the 24 participants, 6 of their injuries were to the upper body and 18 involved the lower body (see Figure 1). Recreationally active was defined as being physically active for at least 20 minutes a day three times per week [24]. Those who were recreation-ally active, who presented with acute pain at the campus recre-ation center athletic training facility, and had a musculoskeletal injury clinically diagnosed by a certified athletic trainer (ATC) within 72 hours following their injury (with the exception of fractures) were included in the study. In addition, all participants were free of any neurological disorder, had no history of diag-nosed psychiatric illness or learning disability (Attention Deficit Hyperactivity Disorder and/or seizures) had no surgical history for at least 6 months, had no existing chronic condition or frac-ture, and were not currently taking any analgesic or non-steroidal anti-inflammatory drugs (NSAIDs) as determined by self-report. Lastly, all participants signed a written informed consent that was approved by the Institutional Review Board.

**Table 1:** Total distribution of recreation athletes by year in higher educa-tion (*n* = 24). Descriptive data indicating most participants were currently enrolled in a master’s program or were a junior in their undergraduate pro-gram. The National Adult Reading Test (NART) was administered during the initial testing session to predict a WAIS-Full Scale IQ score using the following equation: 128-0.83 × NART error score.

	*n*	%	*NART IQ*
Matser’s	7	29%	111.5
Senior	5	21%	110.7
Junior	7	29%	108.6
Sophomore	3	13%	107.2
Freshman	2	8%	113.1

Data was initially collected on 39 participants. Four partici-pants were excluded due to failure to return for follow up testing (*n* = 35). After reviewing the data set for exclusion criteria using the medical health questionnaire, ten participants were re-moved from the data set (*n* = 25). Two of these ten partici-pants who were removed sustained another injury between test-ing points, six were currently taking pain medication on follow up testing, one was previously diagnosed with a mental illness, and one scored above 4 mm on the VAS on follow up testing indicating pain was still present.

Lastly, one participant was removed from the entire data set due to consumption of 200mg of caffeine prior to testing. Previ-ous studies looking at the effects of caffeine have typically seen enhancements in attention at 200-250 mg [8,25]. However, the relationship between caffeine and cognition is affected by many factors including caffeine tolerance, time of consumption, task at hand, personality factors, etc [8,25]. Therefore, because the relationship between caffeine and cognition is not fully under-stood, those who ingested less than 200 mg were included in the data set (*n* = 24).

### 2.2. Protocol

Prior to beginning data collection, all participants completed a health questionnaire that examined previous injuries and over-all health [26]. In addition, acute pain was assessed using the Vi-sual Analog Scale (VAS) [27]. The participant was told to mark his or her current pain intensity on a 100 mm scale. The VAS defines “no pain” as 0-4 mm, “mild pain” as 5-44 mm, “moder-ate pain” as 45-74 mm, and “severe pain” as 75-100 mm. Next, the neuropsychological battery was administered with the fol-lowing tests: the National Adult Reading Test (NART) [7,28], the Digit Span (DS) [7] subtest from the Wechsler 1989, the Rey Auditory Verbal Learning Test (RAVLT) [7,28] and Trail Mak-ing Test B (TMT-B) [7,29]. Participants returned for follow up testing when they believed that they were no longer in acute pain. To confirm participants’ report of not experiencing pain, the VAS was administered at the follow up to determine if the participant was currently not experiencing pain. If so, then the neuropsy-chological battery was subsequently administered. If any par-ticipants reported a pain above 4mm, they were asked to come back within 24-48 hours. The VAS was then administered daily until participants indicated that they were free of any pain. All follow up testing occurred within two weeks of the initial injury. All testing was conducted by a trained a certified athletic trainer with experience in the administration of the neuropsychological battery.

### 2.3. Instrumentation

**NART.** During initial testing the NART was administered to estimate general intelligence and to serve as a benchmark across participants. The NART consisted of 50 phonetically irregu-lar words for the participant to pronounce. The NART scor-ing was performed using a mathematical equation that predicts the Wechsler Adult Intelligence Scale (WAIS-Full Scale) IQ score [26,28].

**DS.** The DS was used to evaluate memory and attention and was broken into two parts: digits forward and backward. In each trial the participant was asked to repeat a span of numbers ei-ther forwards or in reverse order, depending on which portion of the subtest was being administered. The DS starts with only 2 digits with a forward and reverse trial, and progresses up to a span of 8 digits. Once the participant failed to recite the num-bers correctly consecutively two times within the same string of numbers, or once the maximum digit span length was reached (8 forward, 7 backward), testing was terminated. Scoring was based on the number of trials correctly completed forward and backward, which produced an overall score. In this study the digits forward score and digits backward scores were analyzed separately to have a closer look at immediate recall (digits for-ward) versus working memory (digits backward).

**RAVLT.** The RAVLT measures auditory verbal learning and memory. It includes immediate recall, verbal learning, delayed recall, and delayed recognition. It included five trials, each trial consisting of 15 unrelated words (List A). Between each trial the target list was read to the participant at a pace of one word per second [30]. After the fifth trial, an interference list is read [30]. The interference list (List B) consisted of one trial of 15 new words, which the participant was instructed to recite [30]. Af-ter the interference list is recalled, the participant was asked to recall the original words from the first five trials (trial A6), and then again after 20 minutes (trial A7) [30]. Lastly, the partici-pant attempts to recognize as many words from List A as possible from a word set that included distractors [30]. In this study, an alternate RAVLT form was used for the second testing session. Scoring was based on the number of words recalled per trial. Im-mediate memory was derived from the total score from trial A1. Auditory and verbal learning were calculated by the sum of tri-als A1 to A5. Delayed recall post interference list was measured by trial A6. Lastly, delayed recognition was measured using a numerical raw score for recognition list A. Overall, the RAVLT has been noted to have a moderate to low test-retest reliability [28]. Literature indicates the RAVLT may be affected by age and formal education but not gender or intelligence [31].

**TMT-B.** TMT-B was used to measure complex attention, cognitive flexibility, and visual motor tracking. In this investi-gation, only part B was utilized since it is a more complex mea-sure. For this test the participant was given a piece of paper and asked to draw lines to connect consecutive numbers and letters that were circled on the worksheet, alternating between symbol systems, as quickly as possible. This switch between letters and numbers during TMT-B made the test taxing and a measure of executive function due to the complex processing involved with switching between stimuli. Scoring was based on the number of seconds required to complete a task, with a higher score indicat-ing a greater deficiency.

### 2.4. Statistical analysis

The DS was broken into two separate scores: digits forward and digits backward so that immediate recall and working mem-ory could be measured and scored separately. The RAVLT was broken into four scores for a more specific analysis: A1 trial sum, sum of trials A1 through A5, trial A6, and list A recog-nition. Trial A1 measures immediate recall, the sum of trials A1 to A5 measures acquisition, trial A6 measures delayed recall following interference, and List A recognition measures delayed recognition. TMT-B was analyzed using one score: the number of seconds it took to complete the test (see Table 2). The sam-ple of 24 participants was then screened for outliers, which were defined in this study as neuropsychological test scores that were two standard deviations above or below the sample mean. These outliers were removed from the individual tests within the bat-tery. DS forwards, DS backward, RAVLT A1, and RAVLT Sum A1 to A5 had a total sample of 24 (*n* = 24). RAVLT A6 had a sample of 23 (*n* = 23), RAVLT Rec-A had a sample of 21 (*n* = 21), and TMT-B had a sample of 22 (*n* = 22). Seven paired samples *t*-tests were conducted using Statistical Pack-age for the Social Sciences (SPSS) IBM Corp., v.23, Chicago, IL, USA) to compare scores in the pain and non-pain state. A Bonferroni correction was made resulting in an adjusted alpha level of 0.007 due to the multiple comparisons. Effect size was calculated using Cohen’s d. One sample *t*-tests were used to compare the non-pain state to standardized normative data of healthy adults. Lastly, test-retest reliability was calculated using intra-class correlation coefficients with a 95% confidence inter-val (CI).

**Table 2:** Metrics and outcome variables.Metrics and outcome variables that were used to assess the participant when in an acute pain state and again two weeks later when pain free. Numbers in parenthesis indicate the maximum possible score.

Metric	Outcome variable	Score
VAS	Pain intensity	Measured in millimeters (100)

NART	Estimated IQ	128-0.83 × NART error score

DS forward	Immediate recall	Total correct trials (12)

DS backward	Working memory	Total correct trials (12) Sum of recalled words trial A1 (15)

RAVLT A1, A1 to A5, A6, Delayed recognition	Auditory and verbal learning/memory	Sum of words trials A1 to A5 (75) Sum of recalled words trial A6 (15) Sum of words from List A (15)

TMT-B	Executive function (attention, set shifting)	Seconds to complete

## 3. Results

All participants were initially tested within 72 hours of in-jury and follow up tested within two weeks from their injury (8.88 ± 2.5 days). Of the 24 participants, 67% (*n* = 16) re-ported “mild” pain and 33% participants (*n* = 8) reported “mod-erate” pain at the initial testing session (38.02 ± 19.4 mm). All participants reported as “no pain” on their second testing ses-sion (0.67 ± 1.09 mm). The average estimated NART IQ was 110.08 ± 4.49.

Results of the paired samples t-test revealed that when the participants were pain free, their cognitive scores significantly improved on the DS forward and TMT-B. No significant differ-ence was observed for the DS backward, RAVLT A1, RAVLT sum A1 to A5, RAVLT A6, RAVLT recognition list A (see Ta-ble 3). DS forward had an effect size of 0.5 while DS back-ward had an effect size of 0.33. RAVLT A1 had an effect size of 0.20, RAVLT sum A1 to A5 0.01, RAVLT A6 0, RAVLT recog-nition list A 0.82. Lastly, TMT-B had an effect size of 0.79. Re-sults from the one sample t-test showed significant differences between the non-pain state and the normative data for RAVLT A1 and TMT-B (see Table 4). No significant differences were observed for all other measures.

**Table 3:** Variations in cognitive performance during and after a musculoskeletal injury on the Digit Span, the Rey Auditory Verbal Learning Test, and Trails Making Test-B. DSF: Digit Span Forward, DSB: Digit Span Backward, A1: RAVLT A1, SUM: RAVLT Sum A1 to A5, A6: RAVLT A6, REC-A: RAVLT Delayed Recognition list A, TMT-B: Trails Making Test-B. Time point #1 (T1): pain state, and time point #2 (T2) non-pain state. *represents a significant difference between pre-post testing (*p*<0.007)

Subtest	Time	Mean(SD)	N	*p*	Cohen’s d
DSF	1	8.7(1.8)	24	0.001*	0.50
	2	9.6(1.8)	24		

DSB	1	6.5(2.2)	24	0.023	0.33
	2	7.3(2.3)	24		

A1	1	6.4(1.7)	24	0.563	0.20
	2	6.8(2.2)	24		

SUM	1	53.8(7.7)	24	0.953	0.01
	2	53.9(8.3)	24		

A6	1	11.8(2.3)	23	1	0
	2	11.8(2.4)	23		

REC-A	1	14.6(0.7)	21	0.009	0.82
	2	13.8(1.2)	21		

TMT-B	1	48.4(12.6)	22	<0.001*	0.79
	2	39.6(9.4)	22		

**Table 4:** Comparison of the non-pain state to normative data for each subtest of the neuropsychological battery.DSF: Digit Span Forward, DSB: Digit Span Backward, A1: RAVLT A1, SUM: RAVLT Sum A1 to A5, A6: RAVLT A6, REC-A: RAVLT Delayed Recognition list A, TMT-B: Trails Making Test-B.*represents a significant difference between the non-pain state and normative data (*p*<0.05)

Subtest	Ages	Mean difference	N	*p*
DSF*	16-90	0.690	7077	0.001*

DSB	16-90	0.286	6841	0.294

A1	16-19	–0.80	4	0.260
	20-29	–0.100	20	0.857

SUM	16-19	–6.40	4	0.238
	20-29	–1.60	20	0.371

A6	16-19	–0.90	4	0.603
	20-29	0.55	20	0.282

REC-A*	16-19	–1.45	4	0.387
	20-29	–0.750	20	0.027*

TMT-B*	18-24	–6.809	23	0.020*

The results of the intra-class correlations (ICCs) for DSF, DSB, RAVLT sum A1 to A5, and RAVLT A6 indicated a strong positive relationship (see Table 5). The ICC for RAVLT Rec-A indicated a fair relationship, while the ICC for RAVLT A1 was negative, indicating an unreliable measure for this group.

**Table 5:** Intra-class correlation coefficients (95% CI) between pain (T1) and non-pain (T2) states.

Subtest	ICC
DSF	0.837 (0.942 to 0.392)
DSB	0.856 (0.940 to 0. 638)
A1	–0.562 (0.342 to –2.855)
SUM	0.783 (0.906 to 0.491)
A6	0.864 (0.924 to 0.676)
REC-A	0.480 (0.770 to –0.115
TMT-B	0.703 (0.881 to 0.208)

## 4. Discussion

Therefore, the purpose of this study was to explore if the recreationally active individuals who presented with acute pain due to a musculoskeletal injury would have impairments in ex-ecutive functions as measured by the Digit Span, Rey Auditory Verbal Learning Test, and Trails Making Test-B as compared to a non-pain state. The hypothesis of this study was partially met. Improved neuropsychological scores were seen in imme-diate recall and set switching when participants were pain free as measured by the DS forwards and TMT-B. No significance was found between the pain state and non-pain state for working memory in the DS backward or auditory verbal learning mea-sured by the RAVLT, indicating that acute pain does not signif-icantly affect working memory or learning. To our knowledge, this is the first study to examine the effect of acute pain due to a musculoskeletal injury on executive functions in the recreation-ally active population.

Improved neuropsychological scores were seen for DS for-ward and TMT-B. These tests measured immediate recall, atten-tion, and cognitive flexibility. Those that have musculoskeletal injuries have exhibited lower neurocognitive scores compared to controls as measured by the ANAM [13]. The ANAM in-cludes Matching to Sample, which measured spatial and visu-ospatial working memory [13]. While the current study did not directly measure visuospatial working memory, it did measure working memory using the DS backward, which is similar be-cause both tests require the participant to manipulate information while holding it in immediate memory. Contrary to the results of Matching to Sample, these results for working memory did not reach significance. This may be due to visuospatial work-ing memory and auditory working memory are both different facets of working memory. Another subset of the ANAM is the Code Substitution Learning Test, which is similar to TMT-B be-cause it requires visual searching and complex attention to match the numbers and letters in the correct sequence [13]. The cur-rent TMT-B results are similar to previous findings in the Code Substitution Learning Test, indicating a poorer performance post musculoskeletal injury [13].

Improved TMT-B scores were seen when participants were not suffering from acute pain which is consistent with research conducted with chronic pain populations. In a meta-analytical review, those who suffered from chronic pain had a small to moderate impairment in executive function compared to healthy controls [32]. Furthermore, those with chronic pain were slower to complete Trails Making Test A and B [32]. In the current investigation those who were in acute pain were also slower to complete TMT-B compared to those who were not in pain, indi-cating a similar pattern between the acute and chronic pain populations.

In a previous validation study of the Reliable Digit Span (RDS), participants were randomly assigned to be part of a con-trol group, a cold induced pain group, or a simulated pain-related memory impairment group. Typically, an RDS score of 7 or lower is indicative of negative response bias or a lack of effort [33]. RDS scores were calculated by summing the longest for-ward and backward string of digits, leaving the participant with one overall score. Sixty-five percent of the simulated pain group obtained an RDS score of < 7 [33]. Neither the control group nor the pain-induced groups in this study obtained an RDS score below 8 [33]. Results suggest that while sensitive to negative response bias, RDS may be unaffected by acute pain [33]. Al-though the RDS scores from the previously mentioned are cal-culated differently from the DS used in the present study, the results of this study partially coincide with present findings of DS backward scores. The DS used in the present study scored the DS forward and DS backward by summing the total num-ber of correct trials, leaving the participant with two separate scores, compared to the RDS that includes one overall score. Additionally, previous literature has indicated that cold induced pain does not impair working memory or processing speed mea-sured by the Wechsler Adult Intelligence Scale-Fourth Edition [34]. It is important to note that in both studies the authors in-tentionally provoked acute pain in a healthy population, where as in this study, participants were experiencing acute pain due to a physical mechanistic-based injury. Participants who sustain a musculoskeletal injury may suffer from other factors that may affect cognition (ex-fatigue) whereas the healthy population is only experiencing a very temporary pain experience [34]. Re-sults of both studies suggest there are no differences between acute pain and healthy control groups in working memory, pro-cessing speed, and immediate recall [33,34].

No significance was noted for any trials of the RAVLT (A1, sum A1 to A5, A6, or Rec-A). The current results indicate that the pain free group scored lower on the RAVLT than the pain group, which has been previously noted [17]. Our A1 trial score was higher than in a previous study [17]. This may be because participants were tested within the first 0-72 hours of injury.

There may not be enough disruption to the brain to see signif-icant changes in verbal memory and learning within this time frame, or the RAVLT may not be sensitive enough to detect these changes.

All subtests were compared to normative data. The DSF and DSB were compared to normative adult data across 10 samples [35]. The data was collected for 85 years and each of the 10 sam-ples had variable ages. The age range of the normative data was 16-90, and although this is a broad age range it was the only set of normative data that was accessible that examined the same ver-sion of the Digit Span as the current study [35]. The normative DSF and DSB data was calculated using the longest string of cor-rect digits forward and backward, and so the current study calcu-lated the same scores so that they could be compared. The cur-rent study’s DSF average (7.25 ± 0.84) was significantly greater than the normative data (6.56 ± 2.39), indicating that the current study’s sample had better immediate recall ability. This may be due to the very narrow age range and the advanced years of edu-cation of the current sample [35]. Unlike the DSF, the DSB was not significantly different from the normative data. The RAVLT was compared to normative data in age ranges 16-19 and 20-29. The normative data included males and females and was cal-culated using a weighted average mean and standard deviation [36]. This population was reported to have a high average intel-ligence and an average of about 14 years of education, which is congruent with the current study population. The TMT-B was compared to normative data of males and females between ages 18-24 [37]. The average age was 20.17 ± 1.48 with an average education of 12.92 ± 1.01 [37]. The average TMT-B score of the normative data was 48.97 ± 12.69, almost exactly matching the average of the current TMT-B score in the pain state 48.4 ± 12.6 [37]. The non-pain state scored an average of 39.6 ± 9.4, poten-tially indicating that the TMT-B scores during the non-pain state may be due to the practice effect, and/or that the pain intensity was not enough to elicit a deficit in set switching ability.

The results of the test-retest reliability for DSF, DSB, RAVLT Sum A1 to A5, and RAVLT A6 were excellent (see Ta-ble 5) [38]. Test-retest reliability for TMT-B indicated a good relationship, while the relationship for RAVLT Rec-A was clas-sified as fair [38]. Lastly, RAVLT A1 had a negative ICC value, indicating it is an unreliable measure in this study. When look-ing at the data set, there were scores that were both on the high and low ends at both time points, indicating a very variable per-formance of immediate recall. Since the administration of the test was scripted and no other trials of the RAVLT had this same trend, this may be due to the specific population being studied.

When looking at cognitive function, many studies in the acute and chronic pain populations fail to account for psychi-atric disorders, medication use, and the effect of sleep. In addi-tion, many of these studies have a small sample size. This study aimed to control for psychiatric disorders, medication use, and sleep by using a health questionnaire. The questionnaire had an open-ended section for participants to utilize if they felt there was anything else that may affect the study.

Those who were currently taking any type of pain medication were excluded in order to get a more truthful pain score and be-cause improvements in global cognition were observed in those taking analgesics who suffered from chronic pain [12].

This study did not progress without limitations. The lack of a matched control group was a limitation of the study, but was mit-igated by comparing to standardized normative data. Through-out the study, some injuries may have healed faster than others. Therefore, the severity of injury and other factors that may trig-ger pain or re-injury in the two-week time frame was not inves-tigated. The sample population was specific to a convenience sample of those who were recreationally active at a Division I University in the United States and therefore may not be gener-alizable to other populations. There was a smaller sample size than expected based on a power analysis that supported a pop-ulation size of 26 participants. A large effect size was seen for RAVLT recognition list A (*d* = 0.50) although this measure did not reach significance. Interestingly the trend in RAVLT recog-nition list A was in the opposite direction as all other metrics, indicating that participants actually performed better on recog-nition when they were in acute pain. Medium effect sizes were seen for DS forward (*d* = 0.50) and TMT-B (*d* = 0.79). All other metrics resulted in a small effect size. The use of an al-ternate form for the RAVLT was used to control for the practice effect, however there were not alternate forms for DS or TMT-B. It should be noted that previous study reported a significant practice effect for TMT-B of 6.59 seconds in ages 16-29 based on two sessions spaced one week apart [40]. In the current study, the TMT-B had an improved average score of 6.93 seconds on the second trial and was administered 8.88 ± 2.5 days from the initial trial, indicating that the improved scores for TMT-B may be due to the practice effect [40].

Additionally, the pain scores in this study were classified as “mild” to “moderate” which may not have been a strong enough pain intensity to elicit changes in all cognitive domains. Lastly, the neuropsychological battery was not all encompassing due to time constraints and this research did not examine all cognitive domains. A more comprehensive approach with testing may al-low for a differentiation in all executive functions that may be impaired following acute pain.

## Conflict of interest disclosure

No conflicts of interests are reported. The authors declare that the results of this study are honest, clear, and without fabri-cation or falsification.
